# Influence of the relative composition of trace elements and vitamins in physicochemical stability of total parenteral nutrition formulations for neonatal use

**DOI:** 10.1186/1475-2891-11-26

**Published:** 2012-04-17

**Authors:** Bianca W Lobo, Venício F da Veiga, Lúcio M Cabral, Ricardo C Michel, Nádia M Volpato, Valéria P de Sousa

**Affiliations:** 1 Departamento de Medicamentos, Faculdade de Farmácia, Universidade Federal do Rio de Janeiro, Rio de Janeiro, RJ, 21941-902, Brasil; 2 Instituto de Microbiologia Professor Paulo de Góes, Universidade Federal do Rio de Janeiro, Rio de Janeiro, RJ, 21941-902, Brasil; 3 Instituto de Macromoléculas Professora Eloisa Mano, Universidade Federal do Rio de Janeiro, Rio de Janeiro, RJ, 21945-970, Brasil; 4 Faculdade de Farmácia, Universidade Federal do Rio Grande do Sul, Porto Alegre, RS, 90610-000, Brasil

**Keywords:** Total nutrient admixtures, Stability, Lipid globules, Dynamic light scattering, Light obscuration, Optical microscopy

## Abstract

**Objective:**

The present study aimed to evaluate the influence of the relative composition of trace elements and vitamins in physicochemical stability of neonatal parenteral nutrition.

**Material and methods:**

Three formulations for neonatal administration were selected; the main variable was the presence of trace elements and vitamins. The analyses where carried out immediately after preparation and at 24 h, 48 h, 72 h and 7 days after preparation. Three methods were selected to determine globule size: light obscuration, dynamic light scattering and optical microscopy. Complementary evaluation including visual inspection, determination of pH and osmolarity, peroxide levels and measurements of zeta potential were also performed.

**Results:**

There was an observable alteration in color and phase separation in the PN stored at 25°C and 40°C. Neither globule size pattern, nor any other physicochemical characteristic evaluated appeared to be considerably altered in any of the analyzed formulations even after 7 days of storage at 5°C. Globule size in all the PN studied was consistent with the established limit, below 500 nm by DLS measurement, and PFAT_5_ was below 0.05% under all storage temperatures.

**Conclusion:**

Concomitant presence of trace elements and vitamins in the same neonatal formulation did not alter the evaluated aspects of stability.

## Background

Neonates are a special target in parenteral nutrition (PN) practice. Their survival is usually related to its use, particularly if we consider preterm neonates, born before 37 weeks of pregnancy. These patients need differing amounts of nutrients when compared to older children or adults. The concentration of nutrients and factors such as pH directly impacts the physicochemical properties of PN preparations. Pediatric amino acid solutions usually include cysteine hydrochloride, which causes these formulations to be more acidic than adult ones. These neonatal PN formulations present a high proportion of carbohydrates, amino acids and lipids, as neonates have higher energy requirements than older children or adults [1-3]. It is important to consider that the final preparation has a reduced volume when compared to formulations for older children or adults [2], therefore interactions will be increased between substances in PN formulations.

All nutrients necessary to ensure neonatal survival must be contained in a neonatal total PN preparation (TPN). However, incompatibilities may occur when mixing vitamin and trace elements in the same preparation. For these reasons these nutrients are usually administered on alternate days or using two separate bags of TPN. However, administration of indispensable nutrients on alternate days may not have as favorable an outcome as normal nutrition that is provided in a balanced and stable formula. It has been widely recommended that vitamins and trace elements should be administered on the same day, however using two separate bags and infusing each for twelve hours, so as to avoid interactions between these nutrients [4,5]. The same procedure is applied to calcium and phosphorus (Ca/P) neonate supplementation, which must be administrated at a ratio higher than 1.7 Ca:1.0 P, as previously shown by our group [3]. The infusion of two bags daily will increase the number of manipulations necessary when administering a set of TPN thus increasing contamination risks [4,6,7]. Moreover, most services do not compound trace elements and vitamins in the same bag since trace elements could promote oxidation of vitamins. Ascorbic acid is the least stable vitamin and subsequently is oxidized in the presence of oxygen in a reaction catalyzed by copper ions [8]. This reaction leads to two acidic products: threonic acid and oxalic acid, the latter is considerably stronger and could acidify the preparation thus compromising the stability of the emulsion [3,9].

The pH is an important parameter to be controlled when considering lipid stability of a TPN since the presence of positive charges may disturb the efficiency of the emulsifier agent, egg lecithin. A pH value below 5.0 can destabilize the emulsion [9]. Furthermore, trace elements are frequently positively charged and, thus, may alter the distribution pattern of lipid globules when added to a TPN [4,5,9,10].

There are many fundamental aspects to consider regarding the safety of total parenteral nutrition admixtures (TPN) for neonatal use, two of them, sterility and homogeneity of the emulsion, have received special attention in recent years [7,11-14]. Lack of sterility can lead to lethal occurrences. The recommendations for manufacturing sterile products are described in the last edition of the United States Pharmacopeia (USP) [14]. Homogeneity is also a fundamental aspect which is necessary to avoid harmful characteristics in the emulsion. In a stable injectable emulsion, individual lipid particles must be between 0.25 to 0.5 μm in size to maintain a homogenous dispersion in the water solution and be consistent with the chylomicrons size in order to be assimilated by the body [15]. An increase in lipid particle size favors coalescence and creaming which makes the dispersion unstable and potentially dangerous for the patient [11,16]. The USP suggests two methods to measure injectable lipid emulsion (ILE) stability, which include the globule size distribution and the percentage of fat globules (PFAT). To be considered stable and safe, an ILE must meet the USP requirements where mean droplet size cannot exceed 500 nm and PFAT must be less than 0.05 % for PFAT of 5 μm (PFAT_5_) [14]. Many studies have demonstrated that TPN is stable when these parameters are met [12,16-18]. Some authors have also described optical microscopy as an alternative method for characterizing or evaluating physical stability of the PN. These methods use direct observation to determine the size distribution of the internal phase – lipid globules [3,19]. Peroxide levels, zeta potential and visual aspects are additional parameters that can be evaluated when characterizing the physicochemical properties of TPN [16,20]. Nevertheless, until now no official compendium or harmonized methodology has been established to evaluate TPN stability.

In the present study, the effect of added trace elements and vitamins on the physicochemical stability of neonatal TPN formulas was studied. Formulas that contained both of these nutrients were compared to TPN that contained only one of these two components. The impact of these nutrients on the physicochemical stability of neonatal TPN was evaluated by measurement of peroxide levels, pH, zeta potential, osmolarity, sterility and visual aspects at different storage temperatures. Additionally, size distribution of lipid globules was determined by optical microscopy (OM) and the dynamic light scattering method (DLS) [1,21] and the percentage of different sized lipid globules, expressed as PFATx, was determined by light obscuration [12].

## Material and methods

### Composition of the admixtures

Nutrients used in the preparation of the TPN formulations were obtained from laboratories registered in Brazil’s National Health Department. The raw materials had certificates of analysis provided by the suppliers.

The following materials were used for preparation and preservation of the formulations: 10% pediatric amino acid solution with taurine (Primene®) (lots 0400105 and 0401009), 50% glucose solution (lots PR41LO and PR42W9) and water for injection (WFI) (lots PB00X3, PB00X4 and PB01A) from Baxter Laboratories, Brazil; 20% MCT/LCT lipid emulsion (Lipofundin®) (lot 4222A182) and zinc acetate (lot 2513001–2) from BBraun Laboratories, Brazil; 20% sodium chloride (lots 0418957, 0419152, and 0419853), magnesium sulphate (lots 0418736, 0418735 and 0520199 ) and trace-elements (Ped-element®) (lots 0418267 and 0419440) from Darrow Laboratories, Brazil; calcium gluconate (lots 733/02, 736/01, 727/01 and 730/01) from Halex-Istar Laboratories, Brazil; organic phosphate (Phocytan®) (lot 449360-B), from Aguettant, France, vitamin complex (MVI 12 opoplex®) (lot 002/04) from ICN Pharmaceutical, Brazil.

Three formulations denominated NP1, NP2 and NP3, containing vitamins and trace elements were prepared (Table 1). The composition of these formulations was designed based on the worldwide recommendations for nutrients administered by PN in pediatric clinical practice [2,11]. The NP3 formulation, containing both nutrients, is not routinely prepared in the pharmaceutical practice of compounding TPN. This formulation was designed as a model for the present study, to test the influence of the concomitant presence of these nutrients on the final stability of the admixture.

**Table 1:** Composition of the studied TPN

**Nutrients**		**Concentration (volume of stock solution)**		
		**NP1**	**NP2**	**NP3**
Pediatric aminoacids solution 10% (v/v)with taurine		30 mg/mL(30 mL)	30 mg/mL(30 mL)	30 mg/mL(30 mL)
Dextrose solution 50% (v/v)		80 mg/mL(17.3 mL)	80 mg/mL(17.3 mL)	80 mg/mL(17.3 mL)
LCT/MCT lipid emulsion 20% (v/v)		30 mg/mL(15 mL)	30 mg/mL (15 mL)	30 mg/mL(15 mL)
Sodium chloride injection USP		235 mg/mL(1.2 mL)	235 mg/mL(1.2 mL)	235 mg/mL(1.2 mL)
Calcium gluconate injection USP		3 mg/mL(3 mL)	3 mg/mL(3 mL)	3 mg/mL(3 mL)
Organic phosphate		1.2 mg/mL(0.5 mL)	1.2 mg/mL(0.5 mL)	1.2 mg/mL(0.5 mL)
Magnesium sulphate injection, USP		0.15 mg/mL(0.25 mL)	0.15 mg/mL(0.25 mL)	0.15 mg/mL(0.25 mL)
Trace elements (Cu, Cr, Zn, Mn – Ped Element)	Cu	-----	0.2 μg/mL	0.2 μg/mL
	Cr		0.002 μg/mL	0.002 μg/mL
	Mn		0.02 μg/mL	0.02 μg/mL
	Zn		0.5 μg/mL	0.5 μg/mL
			(0.2 mL)	(0.2 mL)
Zinc acetate	-----	3 μg/mL(1.5 mL)	3 μg/mL(1.5 mL)	
Vitamins(MVI 12 opoplex)	A	1.3 μg/mL	-------	1.3 μg/mL
	D	5 μg/mL		5 μg/mL
	E	10 μg/mL		10 μg/mL
	B1	3 μg/mL		3 μg/mL
	B2	3.6 μg/mL		3.6 μg/mL
	B3	40 μg/mL		40 μg/mL
	B5	15 μg/mL		15 μg/mL
	B6	4 μg/mL		4 μg/mL
	B7	60 μg/mL		60 μg/mL
	B9	400 μg/mL		400 μg/mL
	B12	5 μg/mL		5 μg/mL
	C	100 μg/mL		100 μg/mL
		(0.5 mL)		(0.5 mL)
Final volume *q.s.*		100 mL	100 mL	100 mL

The lipid emulsion used in the formulations, containing 20% MCT/LCT, is the most prescribed to neonates in clinical practice of TPN in Brazil and has good stability [1].

It is also interesting to note that the three formulations contained Ca and P, in standard doses, within the same bag.

### Preparation of TPN

Formulations were prepared aseptically, following international recommendations, under a laminar airflow hood in a clean room [7,11,14,22,23] and disposed into ethyl vinyl acetate bags (EVA bags, Baxter Hospitalar Ltda, São Paulo, Brazil). Transfers were performed by the lateral injector using sterile syringes. To attenuate possible incompatibilities, solutions were added to the admixtures according to the recommendations of ASPEN and Food and Drugs Administration - FDA [7,11,22]. Samples were withdrawn aseptically from each mixture at appropriate intervals.

A total volume of 100 mL of each formulation was prepared. For each bag, the theoretical weight was calculated, taking into account the densities of each solution used. After preparation, each bag was weighed and a ± 5% variation was regarded as acceptable [22].

### Sterility assessment

Sterility was assessed by determining the growth of microorganisms in the admixtures. Thus, 5 mL of each TPN were inoculated in flasks containing 4 mL of sterile Trypticase Soy and sterile Trypticase Soy Agar, solidified in the bent position (TSA/TSB). Samples were incubated at 37°C ± 3°C for 14 days, as described by Ribeiro and colleagues [3].

### Physicochemical stability evaluation

All analyses were performed at 0, 24 h, 48 h, 72 h and 7 days after the preparation of the admixtures. The first analysis, time 0, was undertaken at approximately 45 minutes (t_0_ ~ 45 min) after the preparation was made, taking into account the time of sample transportation to the laboratory of analysis. Each TPN admixture was incubated at three different temperatures: 5°C ± 3°C in a refrigerator, to simulate storage conditions, 25°C ± 3°C, room temperature and 40°C ± 3°C, to simulate body temperature. The study was performed with three different lots of each TPN admixture tested (n = 3).

Visual inspection of the emulsion status was performed using the criteria of phase separation and color alteration at 0, 24 h, 48 h, 72 h and 7 days after the preparation of the admixtures, following ASPEN recommendations and a method previously described [3,11]. Digital photographs of the TPN bags were taken at the appropriate time periods and any alterations were documented. The height of the cream layer formed was measured with a 15 cm paquimeter. Before the sampling procedure to the physicochemical analyses, the TPN bags were submitted to a gently agitation in order to guarantee the homogenization.

Theoretical osmolarities of the three admixtures (mOsm/L H_2_Ot) were calculated based on the concentrations of the nutrients added and on the osmolarities described for the solutions used to prepare the TPN. The osmolalities were experimentally confirmed using an Osmette Freezing Point Osmometer (2430 Multi-Osmette, Natick, MA, USA) by the freezing point determination. The osmolality (mOsm/Kg H_2_O_exp_) was converted to osmolarity (mOsm/L H_2_O), using the density of the TPN admixtures measured using a picnometer. The average density obtained was 1.04 at 25°C. The experimental osmolarity of the TPN admixtures was estimated by multiplying the osmolality value obtained by the density of TPNs, subtracted from the concentration of the nutrients in each TPN (0.2 g/L) as described by Martin in 1993 [24].

The pH of the three formulations studied was determined in triplicate for all time points (0, 24, 48, 72 h and 7 days) and at all temperatures studied (5°C, 25°C and 40°C), using a pH meter/conductimeter (Mettler-Toledo, MPC 227, Schwerzenbach, Zurich, Switzerland), calibrated with buffer solutions pH 4.0 and 7.0 (Merck KgaA, Darmstadt, Germany). Samples of 10 mL were collected from each bag for each measurement taken. The pH determination was made by direct immersion of the electrode in the emulsion, at room temperature (25°C).

Zeta potential was determined by microelectrophoresis using a Zetasizer 3000 Hs (Malvern Instruments, Worcestershire, United Kingdom). Samples were injected with 5 mL syringes into the microelectrophoretic cell which was subsequently washed five times with water for injection (WFI; USP) between each measurement. Analyses were made in triplicate, using TPN prepared on different days. A similar sample preparation method as described in DLS analysis below was used for determination of zeta potential.

To determine peroxide levels, 5 mL samples of TPN preparations were transferred into 125 mL Erlenmeyer flasks. Then, 30 mL of acetic acid:chloroform (3:2) was added while stirring to dissolve the sample. Thereafter, 0.5 mL of saturated solutions (1.4 g/ml) of potassium iodide was added and the solutions were stored in a dark place for one minute. Next, a 1% starch solution was added as a colorimetric indicator that confirmed oxidation of iodide to iodine. The iodine that was formed was titrated with 0.01 N thiosulphate volumetric solution.

### Globule size determination

Lipid physical stability was assessed by optical microscopy (OM) and dynamic light scattering (DLS), as described below. The percentage of fat globules (PFATx) was estimated by light obscuration (LO). The determinations followed the same protocol (time and storage temperatures) used in the physicochemical assessments.

#### Optical microscopy (OM)

To determine the size and microscopic characteristics of the lipid globules, the TPN emulsions studied, were evaluated with an optical microscope Axoplan 2 (Carl Zeiss, Jena, Germany) and then switched to a photo camera Color View XS (Soft Imaging System, Münster, Germany), a computer and a TV monitor. An amplification of 100x was used for imaging (Pan Neofluar, Carl Zeiss) with a numeric opening of 1.30 and ∝ / 0.17. The diluent used was the same admixture of TPN without the addition of lipids. Many dilutions were tested in order to obtain optimal microscopy photographs that allowed counting of the lipid globules. After the sample was diluted 5X, an aliquot of 5 μL was dispersed over the lamina. Use of a small volume reduced the amount of Brownian motion within the emulsions and assisted in obtaining clear photographs for posterior analysis.

The OM images were treated using the software AnalySIS (Soft Imaging System, Münster, Germany), that transformed the images obtained into a binary system, so that the particles could be counted and their diameters measured. Approximately, 1000 particles for each field were used for the counts

#### Dynamic light scattering (DLS)

The samples, 1 mL, were collected from the TPN bags, under laminar flow hood, in a clean room, with sterile syringes and needles and appropriately diluted with filtered WFI (0.2 μM) 1:500 to obtain the Kcps (a measure of the sample concentration, obtained by the equipment) from 250 to 350. Each analysis was performed in triplicate, using TPN prepared on different days. DLS measurements were carried out using a *Zetasizer* 3000Hs (Malvern Instruments, Worcestershire, United Kingdom), which uses a helium-neon laser light and an integrated analysis software. The temperature was adjusted to 25°C and the scattering angle was set to 90°, in the DLS equipment, before measurements were taken. The chosen data analysis method was the monomodal or cumulant analysis [25]. The data were expressed as z-average and polydispersity.

#### Light obscuration (LO)

We followed the methodology previously described [17,26,27]. Analysis were performed using the previously calibrated PAMAS Particle Counter equipment (model SVSS, PAMAS GmbH, Sttutgart, Germany), utilizing a sensor HCB-LD-50/50, in extinction mode and software according to CFR 21 part 11. On each day of analysis, 1 mL samples were collected from the TPN bags with sterile syringes and needles and appropriately diluted with WFI 1:200. Next, the diluted samples were transferred to flasks containing magnetic stir bars for analysis. The diameters selected for the analysis were 2.0, 5.0, 7.5, 10, 15, 20, 25 and 30 μm; the flow rate was 10 mL/min and the volume accuracy was ± 0.4 %. The calculation of PFATx was based on previous descriptions [27,28]. Particle sizes were grouped with their respective counts. Data were transferred from PAMAS SVSS software to Microsoft Excel for calculation of PFATx (X = 2, 5 and 10 μm) and each specified diameter was converted on a volume basis [27].

## Statistical analysis

Continuous variables were expressed as mean ± SD. Data obtained by physical and physicochemical assessments were analyzed by paired *t* tests of dependent groups for all TPN and by one way ANOVA. Statistical tools of *GraphPad® Prism* were used in data analysis. Graphical representations were plotted in *Microsoft Excel®* and in *GraphPad Prism®*.

## Results and discussion

### Sterility maintenance and osmolarity evaluation of TPN

Microbial contamination was not detected in any of the formulations under investigation even after storage for seven days at 40 ± 3°C. This test confirmed that sterility of the formulations was maintained and that compounding was adequate, thus ensuring that the results were not altered due to microbial contamination.

Theoretical osmolarity was calculated for the formulations and found to be 875 mOsm/L H_2_O. This value was experimentally confirmed by measuring osmolality for each admixture (mOsm/Kg of H_2_O): 1029 ± 29 for NP1; 1009 ± 14 for NP2; and 1020 ± 32 for NP3. Osmolality was then converted into osmolarity, giving the following values: 864 mOsm/L, for NP1; 845 mOsm/L, for NP2; and 857 mOsm/L, for NP3. It is crucially important to determine osmolarity, as it will define the access of the infusion – central or peripheral – and also whether it is possible to prepare the formulation according to the prescribed concentrations. The osmolarities determined for NP1, NP2 and NP3 comply with the recommendations of ASPEN, which correspond to values below 1200 mOsm/L for central infusion in pediatric patients [7,11].

### Visual inspection and peroxide formation in TPN

Visual inspection of a parenteral admixture, which can be a transparent solution or an opaque emulsion, is mainly limited by human visual accuracy. Despite its limitations, visual observation is essential, since it is one of the methods routinely applied for quality assurance of parenteral admixtures and can be used to detect physical signs of instability [9,16,22,23].

Visual alterations that were observed in NP1, NP2 and NP3 admixtures during the sample period were analyzed and presented in Table 2. Each TPN bag that was stored at a different temperature was individually analyzed. During inspection of phase separation, special care was taken to prevent agitation of the bags to avoid homogenization before observations were recorded, which would lead to an erroneous conclusion. Color alteration was characterized when the final color differed from the initial color.

**Table 2:** Visual inspection of NP1, NP2 and NP3 admixtures

**Admixtures**		**NP1**		**NP2**		**NP3**	
**Time**	**T(°C)**	**Color**^**a**^	**Phaseseparation**	**Color**^**a**^	**Phaseseparation**	**Color**^**a**^	**PhaseSeparation**
**0 h**	5	N/A	no	N/A	no	N/A	no
	25	N/A	no	N/A	no	N/A	no
	40	N/A	no	N/A	no	N/A	no
**24 h**	5	N/A	no	N/A	no	N/A	no
	25	**A**	no	**A**	no	**A**	no
	40	**A**	no	**A**	no	**A**	no
**48 h**	5	N/A	no	N/A	no	N/A	no
	25	**A**	**0.1 cm C**	**A**	**0.1 cm C**	**A**	**0.1 cm C**
	40	**A**	**0.1 cm C**	**A**	**0.1 cm C**	**A**	**0.1 cm C**
**72 h**	5	N/A	no	N/A	no	N/A	no
	25	**A**	**0.2 cm C**	**A**	**0.2 cm C**	**A**	**0.2 cm C**
	40	**A**	**0.2 cm C**	**A**	**0.2 cm C**	**A**	**0.2 cm C**
**7 days**	5	N/A	no	N/A	no	N/A	no
	25	**A**	**0.2 cm C**	**A**	**0.2 cm C**	**A**	**0.2 cm C**
	40	**A**	**0.2 cm C**	**A**	**0.2 cm C**	**A**	**0.2 cm C**

^***a***^ N/A = not altered; A = altered; C = creaming.

The initial stage in emulsion breakdown is creaming, which occurs almost immediately upon mixing of the fat emulsion with other chemical constituents, such as electrolytes or vitamins. The presence of a cream layer is visible as an opaque white layer separated from the remaining TPN admixture at the surface of the emulsion. Although the lipid particles in the cream layer are destabilized, the individual droplet identities are generally preserved. An emulsion with a cream layer is an expected consequence of this pharmaceutical formulation and is generally safe for patient administration since it is reversible [26,27].

Visual inspection indicated that slight creaming, readily reversible with gentle agitation, was evident after 48 h of TPN storage at 25°C and 40°C. Considering that the size of the lipid particles remained unaltered during the study, the formation of a cream layer did not affect the micro-structure of the TPN admixtures. It is important to note that at the indicated storage temperature (2°C to 8°C) none of the admixtures presented a cream layer during the stability study. These results are in accordance with those of Driscoll and colleagues [12] and Lee and colleagues [20], although neither of them evaluated pediatric admixtures, which contain higher relative nutrient concentrations and smaller final volumes than adult admixtures.

Regarding the alteration of color, darkening of the emulsion was observed in the first 24 h of the admixtures stored at 25°C and 40°C. Darkening was observed up to 72 h and did not progress further after this time point. Differences in the visual aspects between the three studied formulations were not detected. The color alteration may be due to *Maillard* reaction or to vitamin degradation, however this was not an aim of this study [29]. Color alterations did not affect droplet size measured either by OM or DLS, neither PFAT_5_.

Peroxide formation was not detected in any of the studied TPN, using iodimetric titration after 72 h, neither for those formulations maintained at 40°C or those which presented darkening or cream layer formation.

### pH evaluation

The pH of all the TPN studied (NP1, NP2 and NP3) was at the recommended range, around 5.5 at time zero [13]. After 72 hours, pH variation was less than 0.1 for all conditions. Even the NP3 admixture, which could be prone to incompatibility due to a possible interaction between vitamins and trace elements, did not have any variation in pH value.

It has already been demonstrated that macronutrient concentrations can alter the stability of TPN admixtures [9,10,30,31]. Very low amino acid concentrations can result in a less stable emulsion, due to a decreased buffering effect, which would directly affect the final pH of TPN admixtures. Final pH values lower than 5.0 have been shown to be destabilizing [12,20,26,31]. The commercial intravenous lipid emulsions for use in TPN admixtures have a standard pH range between 6.0 and 9.0, which keep the negative net charge of the lipid globules [13].

### Zeta potential determination

**Figure 1:**
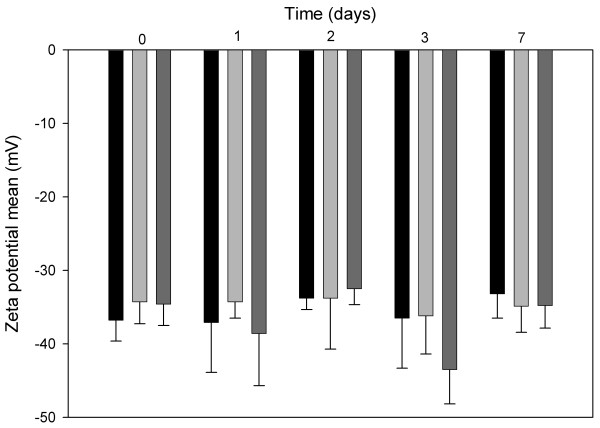
Zeta potential mean variation in NP3, during 7 days of study, at 4°C (black bars), 25°C (light gray bars) and 37°C (dark gray bars) (n = 3; mean ± SD; P > 0.05, between 0 and 7 days).

The means for the NP3 zeta potential for each storage condition are shown in Figure 1. Although slight oscillations were observed at 5°C, 25°C and 40°C during the seven days of study, the results are statistically similar as shown by Student’s *t* test (p > 0.05), for each storage time with respect to time zero. The same pattern was observed for NP1 and NP2. As expected, the zeta potential values, which depend directly on pH, were unaltered, since they were maintained at the recommended range for emulsion stability, between −30 and −50 mV [16,32].

The net surface electrical charge, translated by zeta potential, has been extensively studied previously [16,33,34], nevertheless it has been receiving less attention in recent years. Assessing the zeta potential is critical for quality assurance of TPN formulations, since it translates the efficacy of the emulsifier [35].

### Globule size determination by DLS, OM and LO

**Figure 2:**
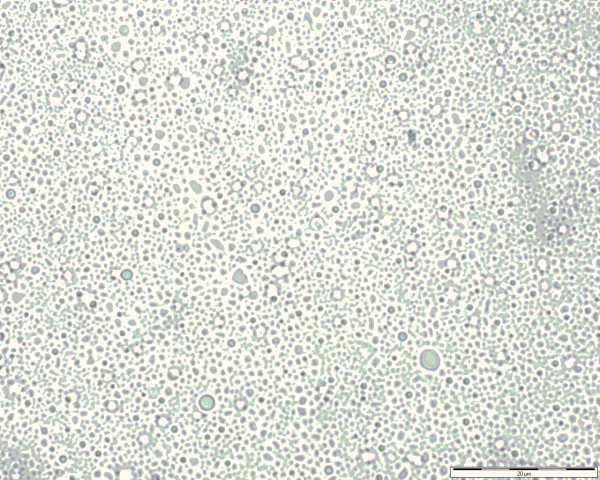
Representative photomicrography of NP3 after 72 h of storage.

Table 3 summarizes the medium diameters of globules obtained by OM (Figure 2), the Z-average and the polidispersity obtained by DLS and the PFAT_5_ (%) determined by LO for NP1, NP2 and NP3, stored at three different temperatures, and time points of 0, 48 h and 7 days.

**Table 3:** **Lipid particles diameter sizes calculated OM, DLS and LO (PFAT**_**5**_**), in NP1, NP2 and NP3**

**Time**	**T (°C)**	**OM**^**a**^** (nm)**	**DLS**		**PFAT**_**5**_**(%)**
			**z-average (nm)**	**PDI**	
**NP1**					
**0 h**	25	810 ± 270	275 ± 24	0.091	0.05 ± 2 · 10^-3^
48 h	5	870 ± 111	269 ± 16	0.085	0.05 ± 1 · 10^-3^
	25	400 ± 250	275 ± 10	0.105	0.03 ± 1 · 10^-3^
	40	800 ± 360	270 ± 01	0.065	0.02 ± 2 · 10^-3^
**7 days**	5	750 ± 250	271 ± 09	0.065	0.04 ± 1 · 10^-3^
	25	560 ± 170	274 ± 08	0.045	0.03 ± 1 · 10^-3^
	40	480 ± 370	272 ± 11	0.055	0.04 ± 1 · 10^-3^
**NP2**					
**0 h**	25	760 ± 320	280 ± 12	0.035	0.02 ± 1 · 10^-3^
48 h	5	950 ± 450	268 ± 30	0.050	0.02 ± 4 · 10^-4^
	25	810 ± 320	267 ± 27	0.065	0.02 ± 6 · 10^-4^
	40	630 ± 240	262 ± 21	0.055	0.01 ± 5 · 10^-4^
**7 days**	5	620 ± 180	257 ± 14	0.045	0.02 ± 8 · 10^-4^
	25	650 ± 240	274 ± 02	0.035	0.01 ± 4 · 10^-4^
	40	540 ± 190	264 ± 14	0.105	0.01 ± 3 · 10^-4^
**NP3**					
**0 h**	25	580 ± 190	272 ± 06	0.053	0.05 ± 1 · 10^-3^
48 h	5	600 ± 180	279 ± 14	0.065	0.05 ± 8 · 10^-4^
	25	650 ± 240	272 ± 05	0.075	0.05 ± 6 · 10^-4^
	40	880 ± 450	274 ± 04	0.065	0.05 ± 5 · 10^-4^
**7 days**	5	520 ± 170	272 ± 06	0.065	0.04 ± 9 · 10^-4^
	25	610 ± 190	268 ± 13	0.060	0.05 ± 1 · 10^-3^
	40	640 ± 210	270 ± 07	0.065	0.05 ± 1 · 10^-3^

number of determinations = 3; results are presented as average ± SD. ^***a***^medium droplet size.

Analysis of DLS values in triplicate gave statistically similar results after application of *t* test (p > 0.05), taking time 0 as reference, and the polidispersity index was less than 0.3 for all samples, as presented in Table 3, showing homogeneity of the dispersions.

The instrument used for particle size distribution analysis capable of detecting a wide range of lipid globule diameters from 1 nm to 3 μm [36]. The results obtained by DLS in this study showed that there was no alteration in the lipid globules diameter at any time or storage temperature employed in this study [37].

The DLS results are in agreement with USP 31 <729 > which points out that the mean droplet diameter size obtained by light scattering methods must be less than 500 nm for lipid injectable emulsions [14,37]. Additionally, the values obtained in this study are similar to those reported by Driscoll and colleagues who used DLS to analyze 34 different parenteral nutrition formulations after compounding, at time 0 and 30 h, the particle diameter varied ± 10 nm [38].

DLS is an efficient, qualitative method that demonstrates the homogeneity of an emulsion, but it fails to quantify different globule diameters that are distant from the mean. In view of this, the USP recommends a second method, LO, to determine the extent of large-diameter droplet tail (> 5 μm) [13,14,18]. This method, also called Light Extinction (LE) employs a technique named single-particle optical sizing (SPOS) and is based on the effect produced by a particle as it crosses a beam of laser light. When the particle (in this case, a globule) crosses a beam of light in the sensing zone, it produces a type of shadow. This causes a voltage pulse in the photodetector, which is converted to particle size. The photodetector is calibrated with polystyrene latex microspheres (PSL) of known size and the voltage pulse that is generated is converted into spherical diameter optical equivalent [39]. LO has been widely studied by some authors in recent years, especially by Driscoll [1,12,13,16,37,38,40] who has been researching intravenous lipid emulsions and parenteral nutrition admixtures for a extended time. Furthermore, LO is recommended by the USP to quantify the volume-weighted percent of fat globules, expressed as the percentage of fat residing in globules larger than 5 μm - PFAT_5_[14,27,41]. USP Chapter <729 > recommends that, to be considered stable, an intravenous lipid emulsions must present PFAT_5_ less than 0.05% [14]. Globules higher than 5 μm can obstruct the lungs, causing emboli, other complications and even death [14,42]. As shown in Table 3, after statistical analysis by one way ANOVA and by *t* test and Wilcoxon test, results show that, for all storage times and at all temperatures studied, none of the formulations presented a PFAT_5_ > 0.05%. Although there were significant differences between NP1 *versus* NP2 and NP2 *versus* NP3, none of the formulations exceeded the limit of 0.05% for PFAT_5,_ established by USP. There were no significant changes observed between NP1 and NP3. The preparation that had the lowest % of PFAT_5_ was NP2, this result may be related to the absence of vitamins in this formulation. The PFAT_5_ results presented in this work are similar to those obtained by Driscoll and colleagues in 2003, when studying the stability of different lipid emulsions for PN admixtures. In that study it was found out that MCT/LCT emulsions added to PN formulations led to more stable admixtures [1,40,43,44]. In addition, PFAT_5_ results corroborate the other results of DLS and OM that will be presented below.

**Figure 3:**
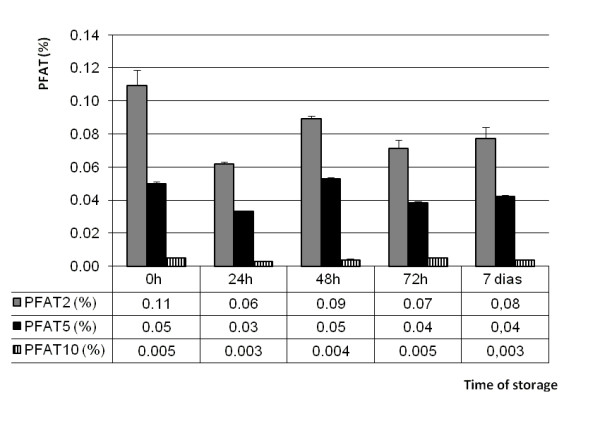
** Relation among PFAT**_**2**_**, PFAT**_**5**_**e PFAT**_**10**_**for NP3, maintained at 5°C ± 3°C (2°C - 8°C), during seven days of study.**

In order to make a comparison, beyond PFAT_5_, the percentage of fat globules were calculated to be 2 μm and 10 μm. Figure 3 presents these percentages for NP3 and confirms that PFAT_5_, did not show significant changes, however PFAT_2_ and PFAT_10_ also remained unchanged as well, These results confirmed that the globule size pattern was maintained at all temperatures studied during the 7 days of storage. In addition, PFAT_2_ and PFAT_10_ were calculated for the formulations NP1 and NP2 and followed the same pattern as shown in Figure 3.

Furthermore, OM was particularly useful for imaging the PN formulations. This technique is a valuable method for the detection of larger droplets and to detect early flocculation in PN formulations [45,46]. However, the size distributions or a medium droplet size in colloidal formulations are not so reliable. For this purpose other methods, like DLS, are more indicated. OM presents poor statistics for PN analysis, as previously demonstrated [13], due the lack of repeatability and accuracy that can be evidenced by the Table 3. As can be observed in Table 3 the medium droplet size obtained by the DLS and OM methods are divergent. However, both methods are complementary and demonstrate the stability of the formulations, as there were not found globules larger than 4 μm in all temperatures tested (Table 4).

**Table 4:** Maximum diameter obtained by OM for NP1, NP2 and NP3 when storage at 25°C (μm)

**Time of storage**	**NP1**	**NP2**	**NP3**
**0 h**	2.66	2.88	1.97
**48 h**	2.88	3.82	3.01
**7 days**	1.97	2.15	1.98

Although, the use of this technique is nowadays easier due to the availability of modern image analyzing software it possesses a major limitation, even presenting a detection limit of approximately 0.2 μm, it is usual to see a diffraction halo around the particle which can lead to gross overestimation of its size [39]. Another significant limitation of this technique is that it is restricted to observation of samples as a whole and is influenced by the sample field observed (Figure 2). The USP indicates OM, in addition to light obscuration, to determine particulate matter in injections [1,13,14,19,22]. Microscope examination is a useful method for measurement of particles from 3 μm to 1 mm and is the only method available to observe and measure individual particles, moreover it is a useful low-cost stability control method and can be easily used is a pharmacy-based compounding unit [39,46]. This can be very interesting in countries such as Brazil, where frequently compounding units cannot invest a large amount of money in equipments.

Table 3 shows the medium diameter values of lipid globules, found in photomicrographs, obtained after analysis of three representative fields for each of the formulations NP1, NP2 and NP3 at times 0, 48 h and 7 days, at all of the studied temperatures. The results obtained for 24 h and 72 h are not shown, but presented the same pattern. Subsequently, results are statistically similar after application of the parametric *t* test (p > 0.05), using time 0 as reference. Even when the overestimation generally associated with this approach is considered, OM analysis confirmed what had already been observed by DLS analysis. The TPN formulations were stable with regard to droplet sizes and homogeneity of dispersion, for every time point and temperature studied. Furthermore, the globules mean diameters were within similar ranges. This pattern was similar to the other formulations, as Driscoll and colleagues previously demonstrated using electron microscopy. They showed that three formulations for pediatric parenteral nutrition, which had a lipid phase MCT/LCT of 20%, maintained the microstructure of lipid globules and were unaltered during 48 h of study [1].

The OM software allowed other parameters, apart from the diameters, to be obtained, such as globule shape, tendency toward sphericity and Ferret diameter. These parameters were not altered for all the formulations, under the different storage conditions, tested in this study.

The results obtained in this study indicate that all the formulations were stable under the studied conditions, with regard to lipid globule size and homogeneity of the dispersions and the concomitant presence or absence of trace elements and vitamins within the formulations.

Notwithstanding, although differences were found in lipid globule diameters, by DLS and by OM, the values were confirmed to be lower than 1 μm by either method tested. Globules measured by OM displayed a medium diameter around 600 nm, while mean diameter sizes obtained by DLS appeared to be around 300 nm. Analysis of images obtained by OM using the software AnalySIS leads to overestimation, as seen above, of globule diameter sizes during conversion to a binary system when a shadow appears around the globules. Because of this OM is only useful when accompanied by and compared to measurements obtained by other methods.

Optical microscopy allowed characterization of globules of higher diameter, which appeared in photomicrographs. Globules were detected in the range of 2 μm, 3 μm, 5 μm and even 10 μm. However, no more than 0.05% of the globules were found with diameter sizes over 5 μm, suggesting that the formulations were stable [1,15,19,20,37,40]. Lewis, in 1993, justified the use of a 1.2 μm line filter for total nutrient admixture infusion showing that catheter’s occlusion by TPN 3 in 1 emulsion is higher than by 2 in 1 emulsions. Additionally other groups, including ASPEN and FDA, demonstrated that catheter’s occlusion was significantly higher in patients that received 3 in 1 TPN therapy, in contrast to those who separately received the lipid emulsion [1,5-7,14,47-49].

No significant differences were found between the formulations in this study. The presence of trace elements and vitamins in the same neonatal formulation, which has a reduced final volume, did not affect the stability parameters evaluated in this study. Color alterations and cream layer formation (reversible) were only noted for the formulations that were stored at 25°C and 37°C. Thus, storage conditions with a temperature between 2°C and 8°C recommended by Brazilian and international guidelines, preserve all the initial characteristics of the admixtures for storage up to 7 days [2,24].

Both DLS and OM are useful methods for the analysis of lipid droplet size in 3 in 1 emulsions of TNP. However, they are complementary procedures to analyze particles of different size ranges. DLS is apparently able to characterize homogeneity of the dispersion and OM, on the other hand, allows visualization of the emulsion with enhanced droplet sizes [50].

According to ASPEN (1998) it is recommended that decisions related to stability and compatibility should be made according to the most reliable information available from the literature or manufacturer of intravenous nutrients. ASPEN recommends that PN formulations should be inspected for signs of gross particulate, contamination and/or phase separation. However, there is no mandatory legislation that obligates pharmacists responsible for compounding TPN to perform physicochemical analysis [11].

Although USP 31 [14] indicates only LO and Light Scattering Methods, as DLS, to determine lipid intravenous emulsion stability it also indicates optical microscopy (OM) for determination of particulate matter in injections [51]. Additionally, British Pharmacopoeia and European Pharmacopoeia indicates both LO and OM, for sub-visible particulate matter determination [13,16,52,53]. DLS and OM appear to be more accessible, considering that most publications recommend that these methods should be applied for quality assurance of TPN formulations and/or lipid injectable emulsions. Visual inspection, pH determination and *zeta* potential constitute complementary methods, although they do not always yield conclusive information [19,20,44,48,54].

## Conclusions

In spite of changes in physical appearance, concomitant presence of trace elements and vitamins in the same neonatal formulation (NP3) did not alter any aspects of stability that were evaluated: pH, peroxide levels, zeta potential, globule size distribution and PFAT_5_, which remained unaltered and in accordance to the limits indicated by the literature. Although changes in physical appearance (color alteration and cream layer formation) were observed, these alterations were not found when the formulations were stored at 2°C and 8°C. Under these conditions, all of the initial characteristics were preserved, even after 7 days. Nevertheless, to ensure the chemical stability of the formulations, a more detailed study is necessary to assess the darkening of preparations and vitamin concentrations, along a time course and over a wide range of temperatures [3,4,8,10,31,48,55].

In view of the results obtained in the present study, the use of 1.2 μm in-line filters is reinforced (to prevent administration of particulate matter to patients. In-line filtration of TPN admixtures has been internationally indicated since the “1994, FDA Safety Alert”. Despite this, the use of these filters may be limited in some countries due the high cost, though its use should be mandatory [23].

## Competing interests

The authors declare that they have no competing interests.

## Authors’ contributions

BWL developed the study design under the supervision of VPS and NMV. VFV performed the microscopic measurements. The LMC and RCM participated on the interpretation of the data. VPS and BWL had primary responsibility for writing the manuscript, but all the others authors read, provided comments on the draft, and approved the final manuscript.
